# Discussion of an environmental depletion assessment method–A case study in Xinjiang, China

**DOI:** 10.1371/journal.pone.0262092

**Published:** 2022-01-21

**Authors:** Zhiping Zhang, Fuqiang Xia, Degang Yang, Yaning Chen

**Affiliations:** 1 State Key Laboratory of Desert and Oasis Ecology, Xinjiang Institute of Ecology and Geography, Chinese Academy of Sciences, Urumqi, China; 2 University of Chinese Academy of Sciences, Beijing, China; Northeastern University (Shenyang China), CHINA

## Abstract

Environmental process assessment based on the environmental depletion index (EDI) is an important part of the long-term monitoring and early warning mechanism of China’s resources and environmental carrying capacity. The EDI aims to realize the unified environmental impact assessment of economic and environmental systems through the ratio relationship between economic growth and pollutant emission growth. However, in terms of pollutant emissions, the EDI ignores the environmental capacity (EC), which means that the effectiveness and objectivity of environmental impact assessment must be verified. In this study, with Xinjiang as an example and based on the EDI, Sulfur dioxide (SO_2_), Nitrogen oxide (NO_x_), Chemical oxygen demand (COD) and Ammonia nitrogen (NH_3_-N) were selected for calculation and assessment both without and with consideration of EC and for discussion of the suitability of the environmental depletion method for resources and environmental carrying capacity. The results indicated that ① the percentages of SO_2_, NO_x_, COD, NH_3_-N and C_EDI_ in counties and cities that tend to be poor and lack EC were 32.98%, 29.79%, 30.85%, 28.72% and 38.30%, respectively, while the percentages in counties and cities with EC were 10.64%, 3.19%, 13.83%, 8.51% and 10.64%, respectively. ② When EC was included, the number of counties and cities where changes in SO_2_, NO_x_, COD, NH_3_-N and C_EDI_ tended to be “poor → good” were 23, 26, 17, 21 and 28, respectively, and the number of counties and cities where such changes tended to be “good → poor” were 2, 1, 1, 2 and 2, respectively. ③ EC inclusion corrected overestimated or underestimated EDI results, making the evaluation results more objective and reasonable. This understanding provides a scientific reference for the coordinated development of the regional economy and environment in Xinjiang and worldwide.

## 1 Introduction

Environmental pollution assessment refers to an objective understanding and evaluation of environmental impact, which is of great significance to put forward reasonable environmental policies, promote the improvement of environmental governance, and realize the coordinated development of economy and environment [1–4]. The objective understanding of environmental pollution is the basis of environmental governance and environmental policy-making [5–11].

Currently, the research on environmental pollution assessment method is one of the key points in the field of environmental assessment. There are a variety of environmental pollution impact assessment methods [12, 13], among which the most representative is environmental impact assessment (EIA), which is considered as a tool to reduce the adverse impact of specific projects and activities. Since the environmental impact assessment in USA was incorporated into the environmental management and protection law by the National Environment Policy Act (NEPA) in 1970, which has been gradually understood and recognized by people [14]. After that, with people’s continuous attentions, environmental impact assessment methods are booming. Among them, life-cycle-assessment (LCA), as a branch of environmental impact assessment, has also been widely used [15]. The LCA emphasizes the environmental impact and resource loss with products as the core, and the regional environmental impact is inevitably ignored [16, 17]. Therefore, with the deepening of research, risk assessment [12, 18], ecological footprint [19], multi-objective linear programming [20], emergy analysis [21], environmental carrying capacity [22, 23] and other methods have been put forward. They either emphasize the comprehensive optimization of ecological, economic and social objectives, or the risk source, or the carrying capacity of natural resources. Some authors also compared and analyzed the regional environmental impact assessment methods, and emphasized the advantages and disadvantages of different methods [12, 13]. In addition, Ana et al. (2014) also summarized the environmental process impact assessment methods, who summarized the current environmental process impact assessment methods into 25 types and divided them into three types: single issue, LCIA intented and process [24]. In general, these methods have made great contributions to environmental impact assessment. At the same time, these methods have also been widely used, so that we have an objective understanding of environmental impact. For example, Tian et al. constructed a coupling model for watershed scale sudden pollution event risk assessment to evaluate the potential risk of risk sources (such as industrial enterprises), the vulnerability of risk receptors (such as drinking water intakes) and environmental risks in different subareas of the basin [18]. Mehzabien et al. recognized the environmental impact of water use in the building life cycle through life-cycle assessment [17].

Environmental depletion index (EDI) is also an important method for environmental impact process assessment. It was proposed by the Chinese government that issued the notice on the “Technical Method for the Monitoring and Early Warning of Resources and Environmental Carrying Capacity (Trial)” (hereinafter referred to as the “Technical Method”) (No. 2043 of the National Development and Reform Commission (NDRC)) [25]. It pursues the unity of environmental system and socio-economic system, depicts the trend of environmental depletion and represents the state trend of environmental carrying capacity. Yu et al. used this method to evaluate the trend of environmental depletion in Beijing [26]. However, as with the above methods, due to different emphasis and insufficient attention to environmental capacity, some special cases are ignored [27, 28]. For example, some regions, which has rapid economic growth, rapid pollutant discharge but no exceed the regional environmental capacity, its environmental impact is overestimated, while some regions with rapid economic growth and slow pollutant emission, but exceeding the regional environmental capacity, its environmental impact is underestimated. EC is the key to connecting the environmental system and socioeconomic system [27, 29–31]. Therefore, we propose that the "environmental capacity" parameter based on the environmental depletion index modify the environmental depletion index in order to solve the above problems. Through the comparative analysis of EDI and modified EDI, the suitability of environmental depletion evaluation method is discussed. EDI is an important part of the monitoring and early warning system of China’s resource and environmental carrying capacity. In-depth discussion and research on its methods is of great significance for curbing environmental deterioration, stabilizing environmental carrying capacity, solving air and water pollution problems and promoting sustainable development [23, 25, 32].

Xinjiang is an ecologically fragile area, and the contradiction between economy and ecology is more prominent and typical. This paper takes Xinjiang as an example, based on the EDI, an environmental process assessment and comparative analysis are carried out both with and without considering EC. First, we evaluate the single index and perform a comparative analysis by decomposing the EDI. Second, pollutants (including air and water pollutants) are integrated to evaluate, and a comparative analysis is carried out. Finally, we analyze the applicability of the method to improve the monitoring and early warning technology system of resources and environmental carrying capacity, improve the scientific basis of policy-making, realize regional sustainable development and promote the construction of ecological civilization.

## 2 Methods and data

### 2.1 Study area

**Fig 1:**
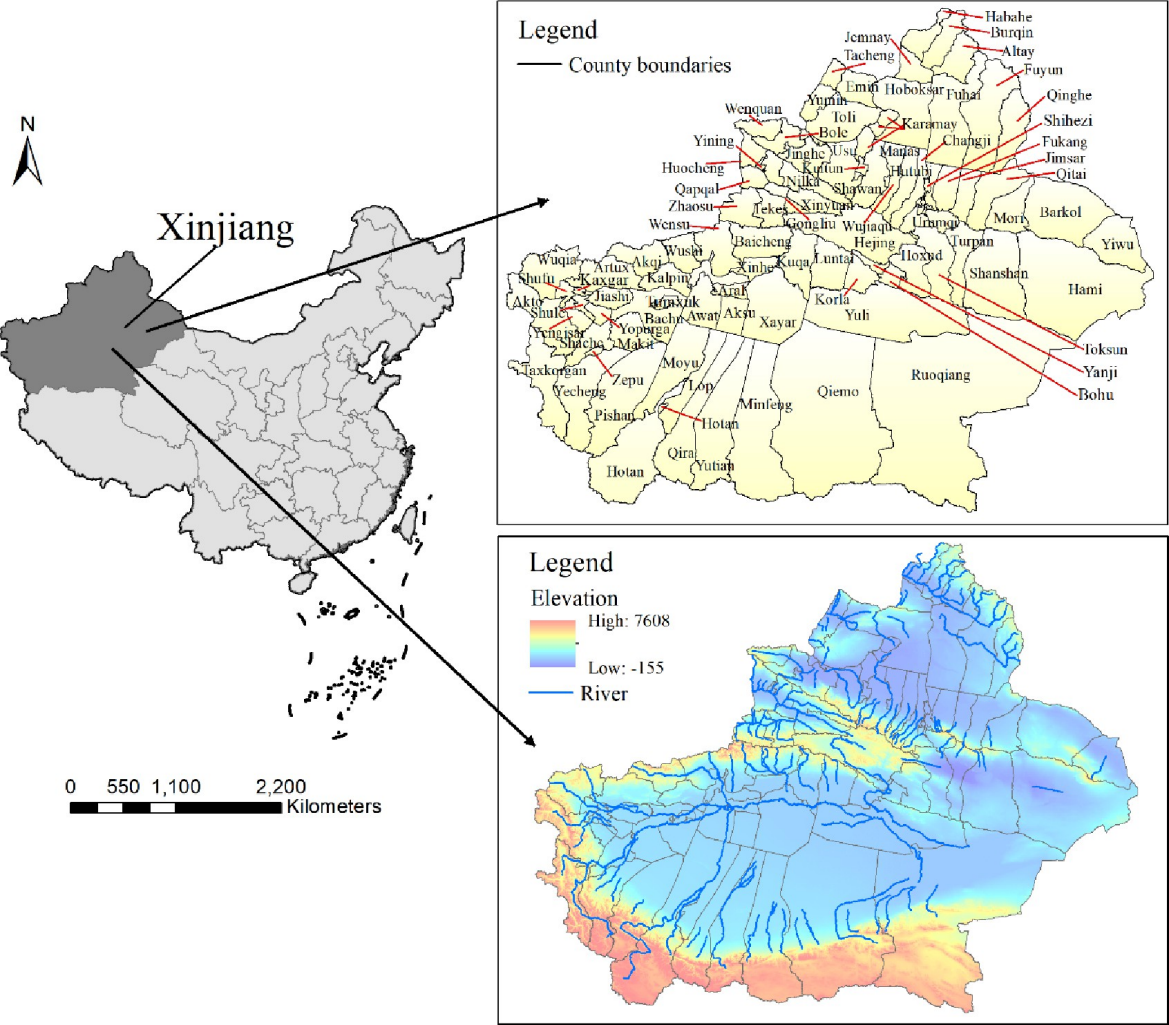
Map of the study area (location map, topographic map) (Based on map sources: GS (2016) 2556).

Xinjiang, China, is located in the middle of the Eurasian continent (34.25°N-49.17°N, 73.33°E-96.42°E) and has a total area of 1 million 665 thousand square kilometers [33]. Xinjiang is far from the ocean. Topographically, it has high elevations in the west and south and low elevations in the east and north. It has a unique landscape of “three mountains surrounding two basins” (Fig 1). Xinjiang has an extremely dry, warm, temperate continental climate, manifested mainly by low precipitation, strong evaporation, long winters, short springs and autumns, large amounts of sunshine, a wide temperature range, and frequent sandstorms. The fragile geographic environment makes the contradiction between social development and the environment more acute. In particular, air and water pollution has existed for a long time [34, 35], for example, in 2019, the water quality of lakes and reservoirs in Xinjiang was slightly polluted, and the proportion of lakes and reservoirs with pollution above a moderate level was as high as 19.3%. Additionally, more than 13.7% of the 14 prefectures in the region had moderate air pollution [36]. Although Xinjiang is located on the northwestern border of China, it has a special strategic position at the leading edge for China to move toward Central Asia, South Asia, West Asia and even Europe. This region is also called the “core area” for the construction of China’s Silk Road Economic Belt [37–40]. Ecological environmental destruction and environmental pollution will directly affect sustainable economic and social development and the implementation of the “Belt and Road” initiative in China [41].

### 2.2 Data sources and processing

The research data mainly include emissions of air pollutants and water pollutants and the GDP data of counties and cities. With the construction of China’s ecological civilization (proposed in 2012) and the launch of environmental protection policies, to compare the effect before and after the policy, 2010–2015 was selected as the study period. The pollutant emission data originate from the Xinjiang Environmental Protection Department, with emission data from 94 counties and cities in Xinjiang. The GDP data of each county come from the Xinjiang Statistical Yearbook [42]. In addition, the standard concentration limits of air pollutants and water pollutants are included, with data from the Environmental Air Quality Standard (GB3095-2012) [43] and the surface water environmental quality standard (GB38382002) [44]. The water pollutant concentration monitoring data come from the Xinjiang Environmental Protection Department. Xinjiang’s administrative vector data come from the national 1:1 million basic geographic database provided by the National Catalogue Service for Geographic Information of China (http://www.webmap.cn/). All the abbreviations used in this study are shown in S1 Appendix.

### 2.3 Methods

Environmental process assessment includes single-index assessment and comprehensive environmental assessment. The single-index evaluation based on EDI, that is, the ratio relationship between economic growth and the growth rate of environmental pollution emissions (section 2.3.1.1). The EC correction method of single-index evaluation means that the relevant EC parameters are included; that is, the ratio relationship between economic growth, environmental pollution emission growth and EC (section 2.3.1.2). Comprehensive environmental evaluation includes both the EDI (section 2.3.2.1) and EDI with EC corrected (section 2.3.2.2). Finally, based on the single evaluations and comprehensive evaluation, the evaluation results based on the EDI and EDI with EC corrected are compared and analyzed.

#### 2.3.1 Single-index assessment

*2*.*3*.*1*.*1 EDI*. The single-index evaluation based on the EDI includes the air pollutants and water pollutants.

**(1) Atmospheric EDI**

According to the “Technical Method” [26], the calculation formula is as follows:

$$A_{EDI}=\sqrt[{n}]{\frac{E_{t+n}}{E_{t}}*\frac{GDP_{t}}{GDP_{t+n}}}-1$$ (1)

where AEDI is the EDI of sulfur dioxide (SO2) and nitrogen oxide (NOx); t is the base year (2010, the same as below); Et and GDPt are the pollutant emissions and GDP of each region in the base year; and Et+n and GDPt+n are the pollutant emissions and GDP of each region in the nth year (2015, the same as below) after the base year. When AEDI < 0, the change tends to be good; when AEDI > 0, the change tends to be poor [22].

**(2) Water EDI**

The calculation formula is as follows:

$$W_{EDI}=\sqrt[{n}]{\frac{E_{t+n}}{E_{t}}*\frac{GDP_{t}}{GDP_{t+n}}}-1$$ (2)

where WEDI is the EDI of chemical oxygen demand (COD) and ammonia nitrogen (NH3-N); t is the base year; Et and GDPt are the pollutant emissions and GDP of each region in the base year; and Et+n and GDPt+n are the pollutant emissions and GDP of each region in the nth year after the base year. When WEDI < 0, the change tends to be good; when WEDI > 0, the change tends to be poor [28].

*2*.*3*.*1*.*2 EDI with EC corrected*. **(1) Atmospheric EDI**

Considering the impact of AEC on air pollution emissions, the analysis of the EC parameter is included in the EDI. The EC parameter is the ratio of the final emission of environmental pollutants to the AEC.


$$A_{EDI}{}^{'}=\sqrt[{n}]{\frac{E_{t+n}}{E_{t}}*\frac{GDP_{t}}{GDP_{t+n}}*\frac{E_{t+n}}{E_{C}}}-1$$ (3)

Et+n, Et, GDPt and GDPt+n are consistent with the above formula. The AEC is calculated using the formula mentioned in the “Technical Method for Making Local Emission Standards of Air Pollutants”:

$$E_{c}=A*C_{k}*\sqrt[{2}]{S}*\alpha$$ (4)

where A is the total quantity control coefficient, calculated based on the standard compliance rate of 90%, and A is 7.14 in Xinjiang; Ck is the annual average concentration limit of environmental pollutants under the environmental quality standards [29]; S is the total area of a county or city; and α is the allowable emission factor of the pollution sources, which is 0.15.

**(2) Water EDI**

Considering the impact of WEC on water pollutant emissions, the EC parameter is included in the EDI analysis. Because the calculation of the EC of water pollutants involves many factors and the calculation process is complex, the annual average pollutant concentration of monitoring points is used to replace the calculation. The specific formula is as follows:

$$W_{EDI}{}^{'}=\sqrt[{n}]{\frac{E_{t+n}}{E_{t}}*\frac{GDP_{t}}{GDP_{t+n}}*\frac{W_{t+n}}{W_{s}}}-1$$ (5)

Et+n, Et, GDPt and GDPt+n are consistent with those of the above formula. Wt+n is the final average concentration at the monitoring point of the area, and Ws is the concentration under the water discharge standard.

*2*.*3*.*1*.*3 Comparative analysis*. The suitability of EDI evaluation, which includes EC, is discussed through comparative analysis. The comparative analysis consists of two parts: one is the statistical comparative analysis of the results of the two methods, and the other is the analysis of the causes of the two methods. The first part of the paper is based on Table 1. The second part analyzes the causes of the above formula resolution. The atmospheric EDI is decomposed according to formulas (1) and (3), and the water EDI is decomposed according to formulas (2) and (4). The causes of decomposition are categorized into three parts: a pollutant emission growth index, an economic development index and EC. $\frac{E_{t+n}}{E_{t}}=PEI$ is the pollutant emission growth index, $\frac{GDP_{t}}{GDP_{t+n}}=ED$is an economic development index of a county or city, $\frac{E_{t+n}}{E_{c}}=AEC$ denotes the AEC, and $\frac{W_{t+n}}{W_{s}}=WEC$ denotes the WEC.

**Table 1:** Comparison of the two results (statistics of the number of counties and cities with four change patterns of pollutants).

Type of change	Definition
**Poor → good**	A_edi_>0 → A_edi_’<0 or W_edi_>0 → W_edi_’<0
**Poor →poor (unchanged)**	A_edi_>0 → A_edi_’>0 or W_edi_>0 → W_edi_’>0
**Good → poor**	A_edi_<0 → A_edi_’>0 or W_edi_<0 → W_edi_’>0
**Good →poor (unchanged)**	A_edi_<0→ A_edi_’<0 or W_edi_<0 → W_edi_’<0

#### 2.3.2 Comprehensive environmental assessment

*2*.*3*.*2*.*1 EDI*. Based on the evaluation results of the four pollutants in the single-index evaluation (section 2.3.1.1) of the EDI, the comprehensive EDI (C_EDI_) is classified. On the one hand, based on the formula, the threshold standard is logically judged to be 0; that is, when the annual average change in pollutant emissions per unit of GDP is negative, the change tends to be good, and the classification should be good. When the annual variation in pollutant emissions per unit of GDP is positive, the classification should be worse. On the other hand, combined with the “Technical Method”, and the comprehensive classification standard proposed by Yu et al. [28], the classification standard of C_EDI_ in this paper is shown in Table 2.

**Table 2:** Classification standard of comprehensive environmental depletion indexes.

Description	Trend	Classification standard
**Comprehensive environmental depletion index**	Poor	The growth rate of 2 kinds of speed indexes is higher than 0
	Good	Circumstances other than those mentioned above

*2*.*3*.*2*.*2 EDI with EC corrected*. The comprehensive EDI classification based on EC correction (C_EDI_’) is consistent with the comprehensive EDI classification based on the EDI, and C_EDI_’ is also obtained based on Table 2.

*2*.*3*.*2*.*3 Comparative analysis*. Since the four pollutants have been decomposed into a single index, to avoid repeated analysis, only the comparative analysis of the evaluation results of the two methods is performed in the comprehensive environmental assessment.

## 3 Results and discussions

### 3.1 Single-index assessment results

#### 3.1.1 EDI

**Fig 2:**
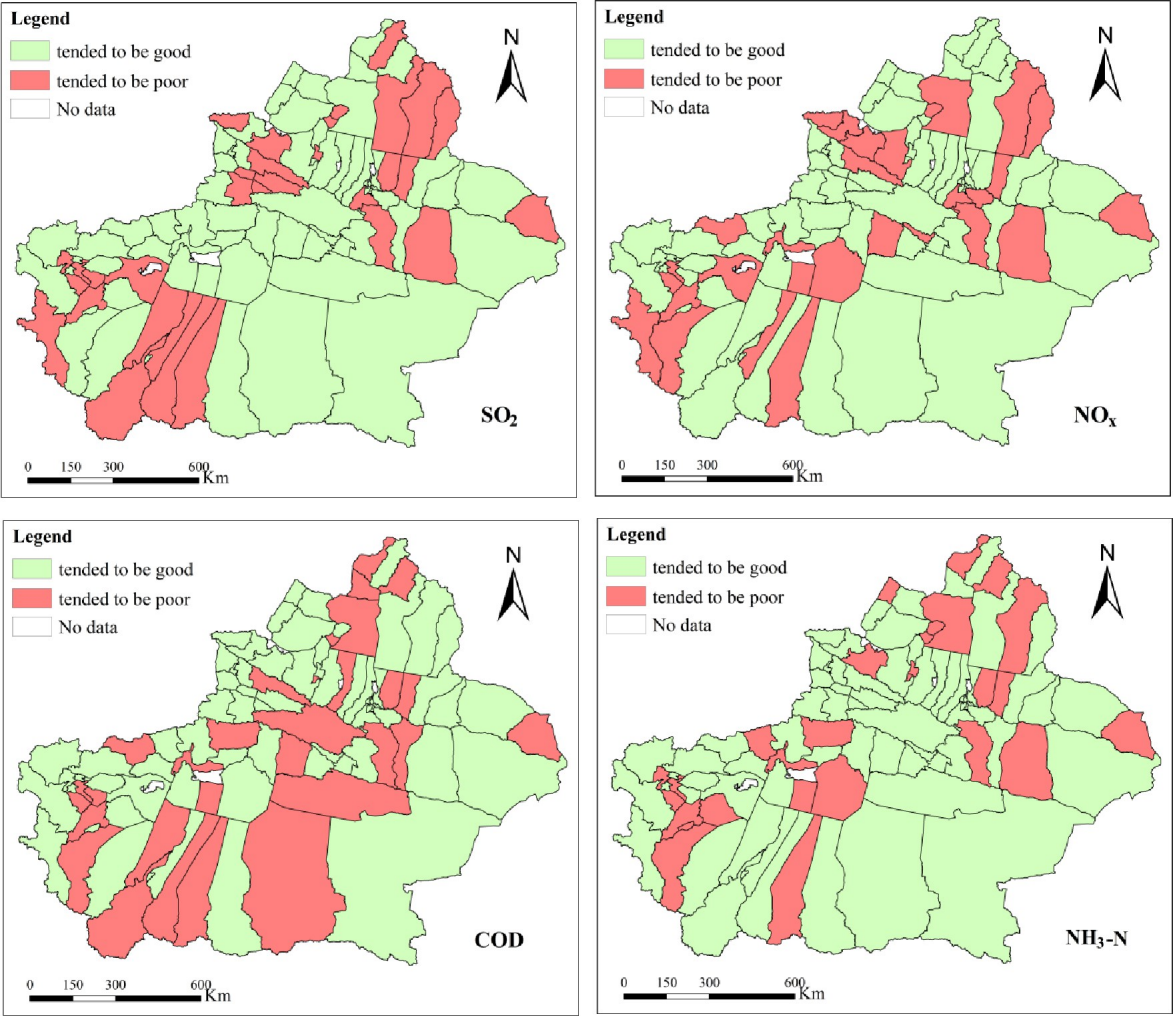
Single-index assessment based on EDI.

According to formula (1), the A_EDI_ of 94 counties and cities of Xinjiang is shown in Fig 2. SO_2_ tended to be poor in 31 counties and cities, accounting for 32.98%, and tended to be good in 63 counties and cities, accounting for 67.02% (Fig 2 –SO_2_). NOx tended to be poor in 28 counties and cities, accounting for 29.79%, but tended to be good in 66 counties and cities, accounting for 70.21% (Fig 2 –NO_x_).

According to formula (2), the W_EDI_ of 94 counties and cities is shown in Fig 2. Among them, the COD tended to be poor in 29 counties and cities, accounting for 30.85%, and tended to be good in 65 counties and cities, accounting for 69.15% (Fig 2 –COD). The NH_3_-N tended to be poor in 27 counties and cities, accounting for 28.72%, and tended to be good in 67 counties and cities, accounting for 71.28% (Fig 2 –NH_3_-N).

#### 3.1.2 EDI with EC corrected

**Fig 3:**
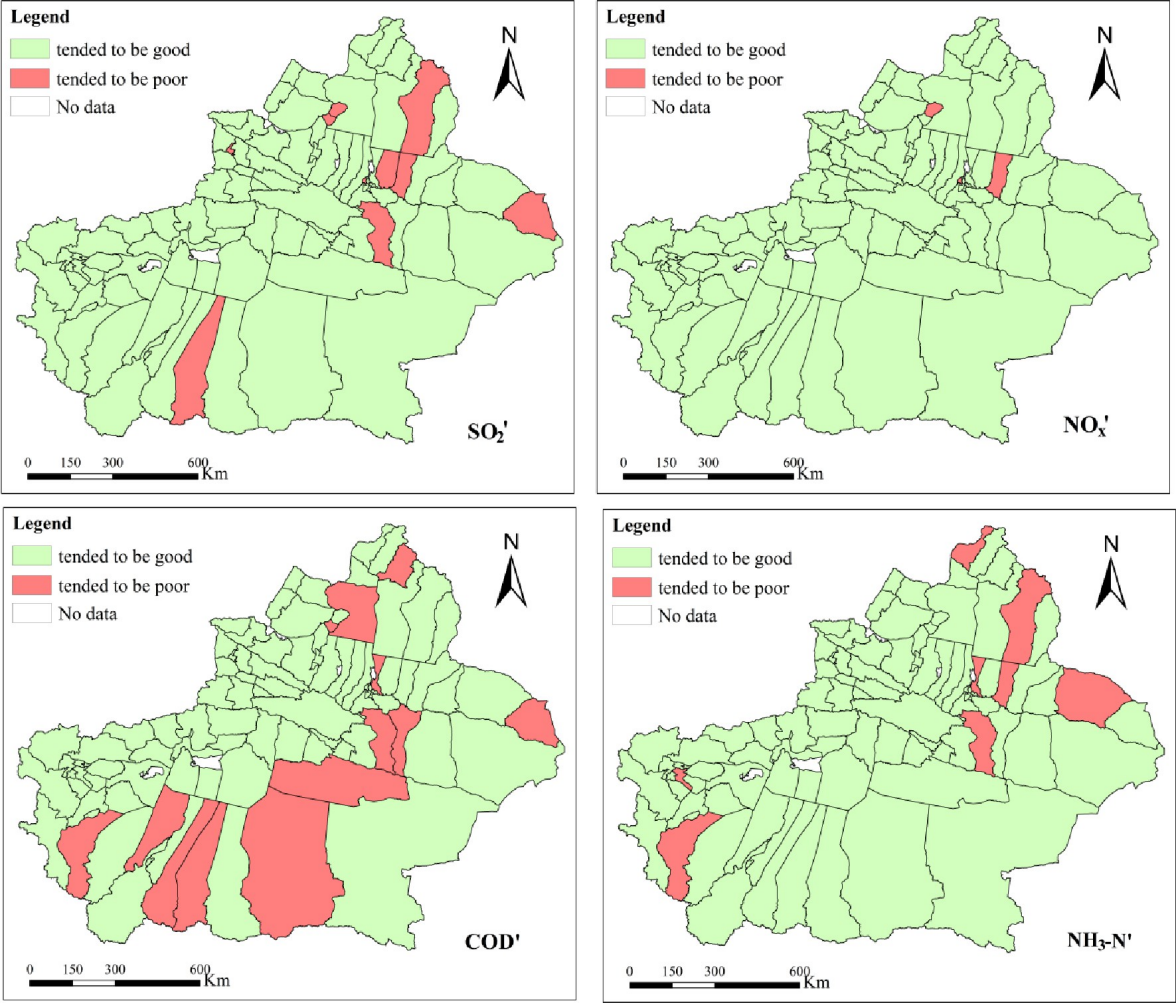
Single-index assessment results based on EDI with EC corrected.

According to formula (3), the AEDI’ with consideration of EC of 94 counties and cities is shown in Fig 3. The SO2 with consideration of AEC tended to be poor in 10 counties and cities (Fig 3 –SO2’), namely, Tou Tunhe district, Bai Jiantan district, Urhe district, Toksun county, Yiwu county, Fukang city, Jimsar county, Yutian county, Yining city and Fuyun county, accounting for 10.64%, and tended to be good in 84 counties and cities, accounting for 89.36%. The NOx with consideration of AEC tended to be poor in 3 counties and cities (Fig 3 –NOx’) namely, Tou Tunhe district, Urhe district and Jimsar county, accounting for 3.19%, and tended to be good in 91 counties and cities, accounting for 96.81%.

According to formula (5), the W_EDI_’ of the counties and cities of Xinjiang is shown in Fig 3. The COD with consideration of WEC tended to be poor in 13 counties and cities (Fig 3 –COD’), namely, Midong district, Bai Jiantan district, Turpan city, Toksun county, Yiwu county, Yuli county, Qiemo county, Yecheng county, Moyu county, Qira county, Yutian county, Hoboksar Mongol Autonomous County and Altay city, accounting for 13.83%, and tended to be good in 81 counties and cities, accounting for 86.17%. The NH_3_-N with consideration of WEC tended to be poor in 8 counties and cities (Fig 3 –NH3-N’) namely, Midong district, Toksun County, Barkol Kazak Autonomous County, Jimsar county, Shule county, Yecheng county, Fuyun county and Habahe county, accounting for 8.51%, and tended to be good in 86 counties and cities, accounting for 91.49%.

#### 3.1.3 Comparative analysis

**Fig 4:**
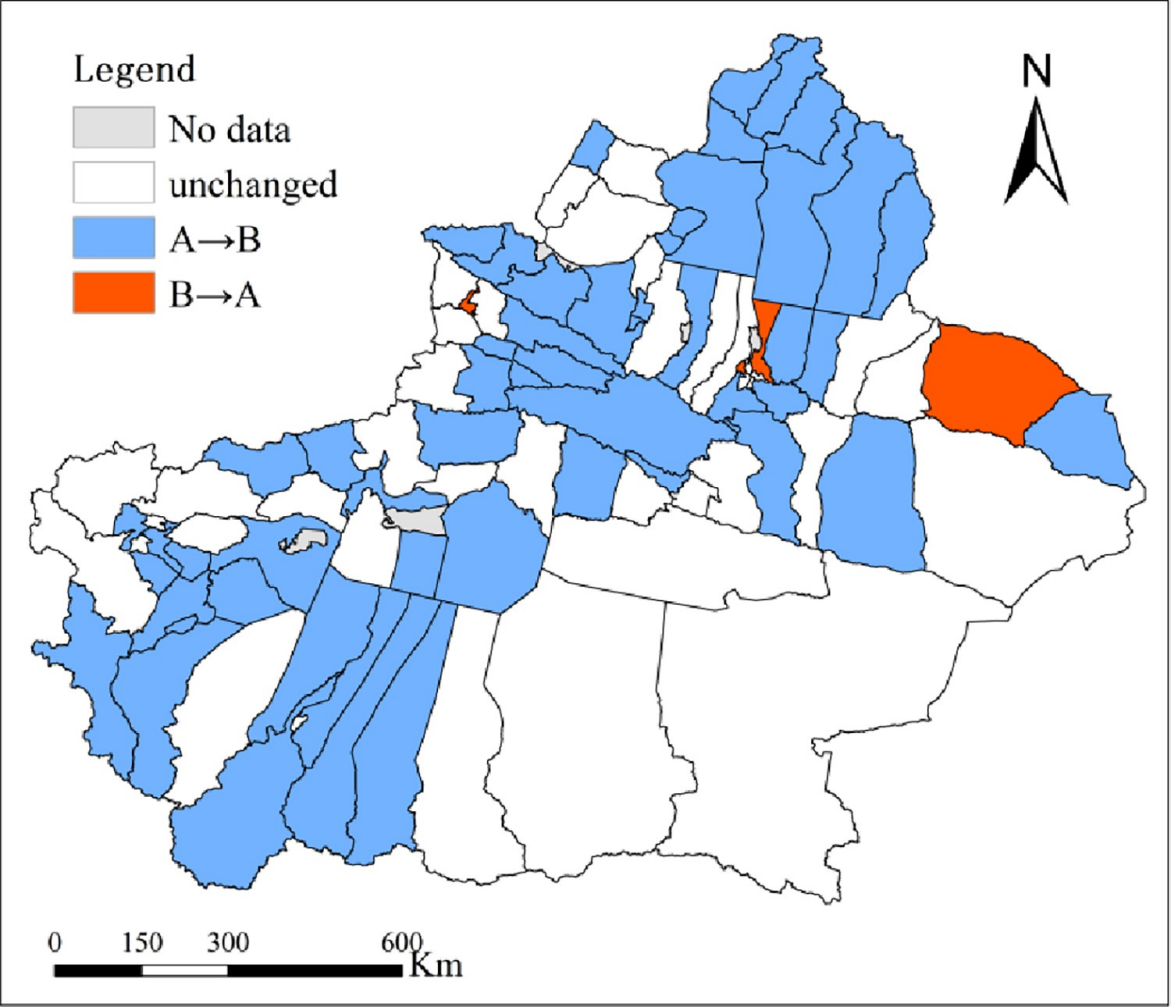
Quantitative and visualized analysis of changes in 4 kinds of pollutants based on the EDI and EDI with EC corrected (A→B refers to the change trend from poor to good, and B→A refers to the change trend from good to poor, as in Fig 9).

According to section 2.3.1.3, the number of county and city changes in the EDI of the four pollutants with/without considering the EC are shown in Table 3. The results indicate that with consideration of EC, the numbers of counties and cities where SO_2_, NOx, COD and NH_3_-N remained unchanged were 69, 67, 76, and 71, respectively. The numbers of counties and cities where changes in SO_2_, NO_x_, COD and NH_3_-N tended to be “poor → good” were 23, 26, 17 and 21, respectively, and the numbers of counties and cities where such changes tended to be “good → poor” were 2, 1, 1 and 2, respectively. This finding indicated that the environmental assessment results of over 1/4 of the counties and cities changed with consideration of EC. These changes tended to be “good → poor”, mainly in Midong district, Barkol Kazak Autonomous County, Tou Tunhe district and Yining city (Fig 4 –“B→A”). The changes tended to be “poor → good” in Fuhai county, Fuyun county, Habahe county, etc. (Fig 4 –“A→B”).

**Table 3:** Comparative analysis of changes in counties and cities based on the EDI and EDI with EC corrected.

Trend	SO_2_	No_x_	COD	NH_3_-N
**Poor → good**	23	26	17	21
**Good → poor**	2	1	1	2
**No change**	69	67	76	71

**Fig 5:**
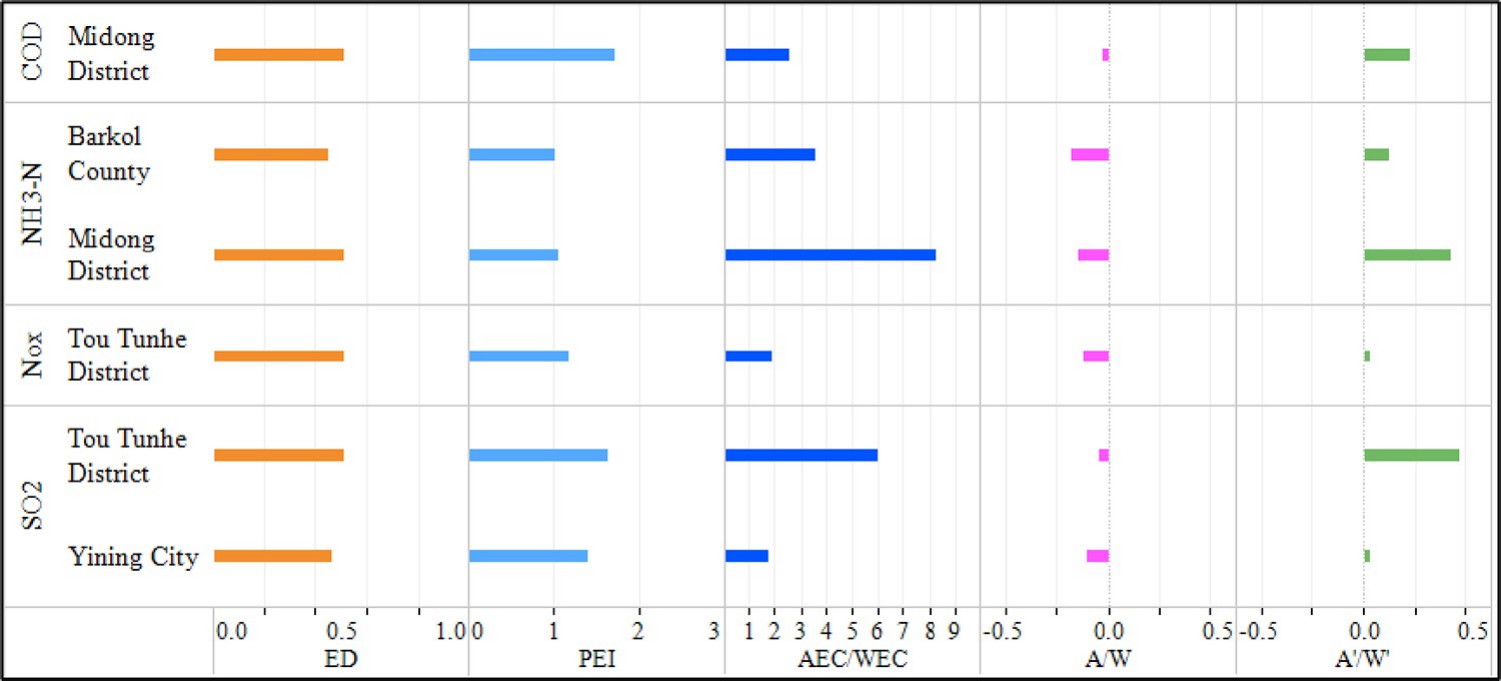
Causes of decomposition of pollutants in counties and cities with a “good → poor” change trend (A is A_EDI_, W is W_EDI_, A’ is A_EDI_’ and W’ is W_EDI_’, as in Figs 6 and 7).

According to section 2.3.1.3, the causes of decomposition of the four pollutants in “good → poor” counties and cities are shown in Fig 5, which shows that the economic development levels of Midong District, Balikun County, Tou Tunhe district and Yining city are all approximately 0.5 (Fig 5 - ED), and the pollutant emission growth index is between 1 and 2 (Fig 5 –PEI). Regardless of the AEC/WEC, the assessment results of the environmental depletion of 4 kinds of pollutants based on the EDI were manifested as “good” (Fig 5 –A/W). However, with consideration of EC that was greater than 1 (Fig 5 –AEC/WEC), far beyond the allowable environmental standards, the assessment results of the environmental depletion of 4 kinds of pollutants were manifested as “poor” (Fig 5 –A’/W’). Regardless of EC, this resulted in negative assessment results for environmental depletion (that is, the environment changed in a negative direction) being deemed positive so that the regional environment was underestimated and unrewarded. After EC was taken into account, the underestimated environmental depletion assessment results of these counties and cities were corrected.

**Fig 6:**
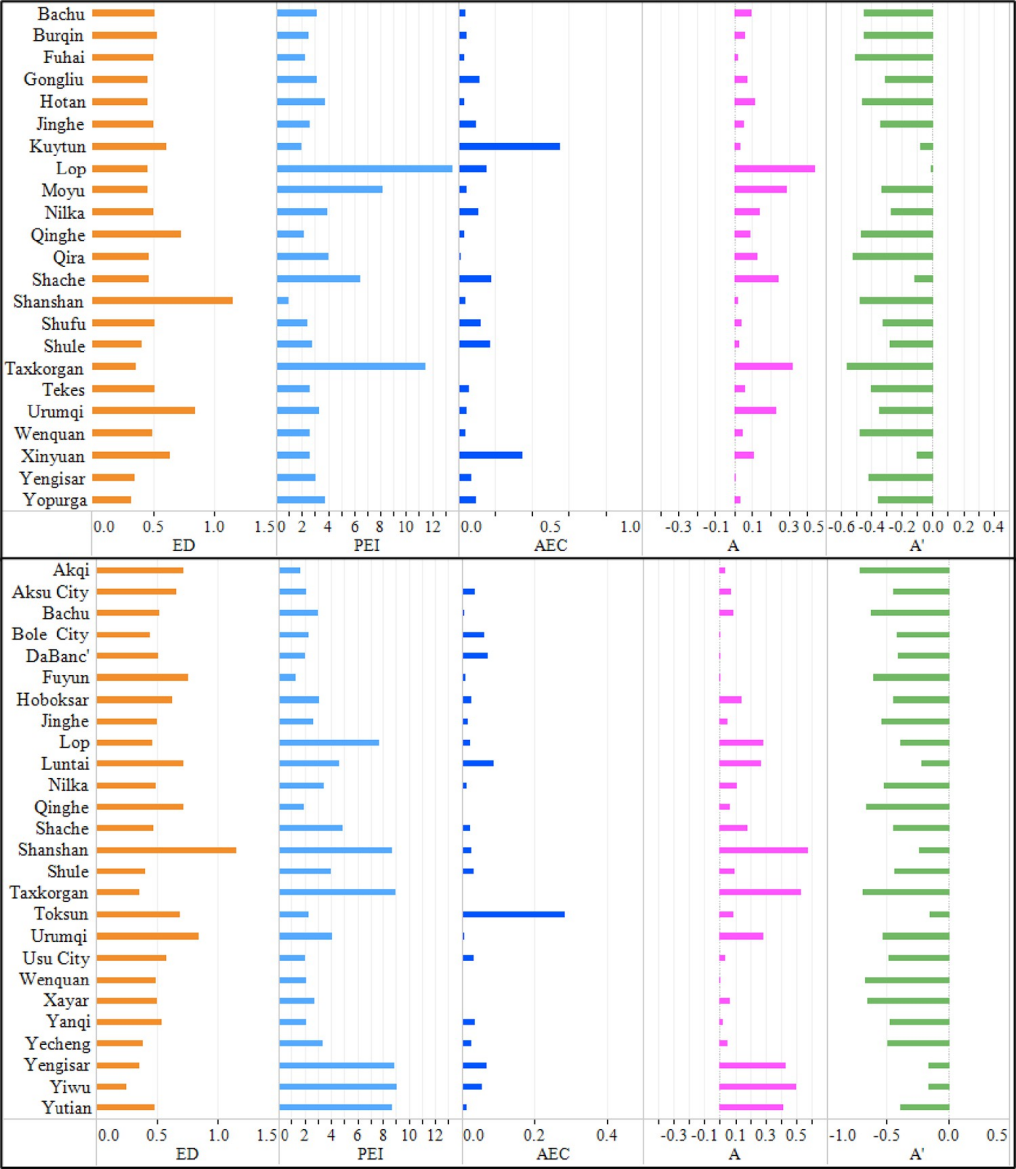
Causes of decomposition of air pollutants in counties and cities with a “poor → good” change trend.

**Fig 7:**
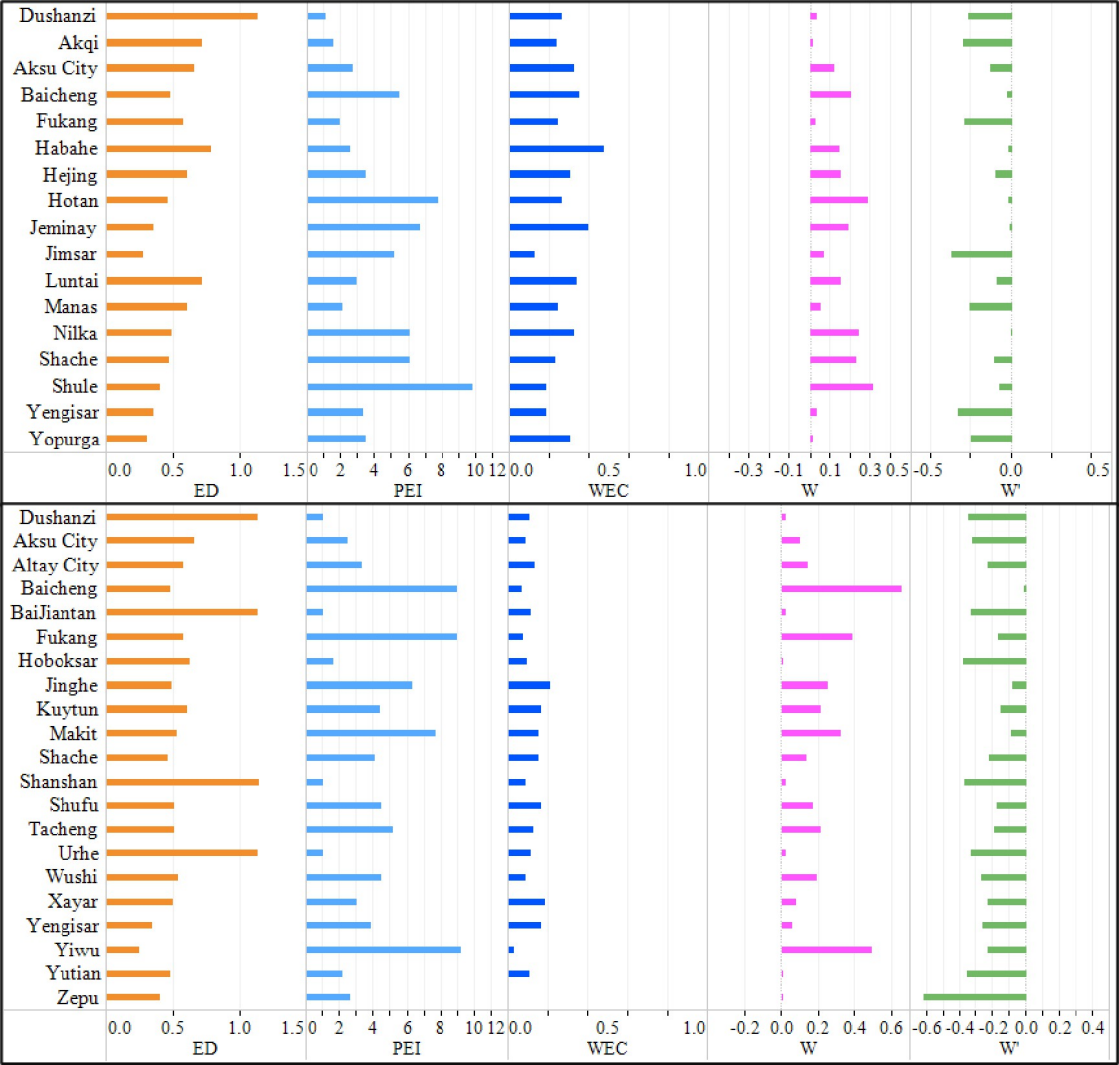
Causes of decomposition of water pollutants in counties and cities with a “poor → good” change trend.

According to section 2.3.1.3, the cause of decomposition of the four pollutants in the counties and cities with a “good → poor” change trend is shown in Figs 6 and 7. The economic development index of these counties and cities was mostly 0.5, and the pollutant emission growth index was mostly greater than 2. When EC was not considered, the assessment results of the environmental depletion of 4 kinds of pollutants based on the EDI were manifested as “poor”. However, with consideration of EC that was smaller than 1, not far beyond the allowable environmental standards, the assessment results of the environmental depletion of 4 kinds of pollutants were manifested as “poor”. If EC is neglected, the positive assessment results of environmental depletion (that is, the indication that the environment is changing for the better) were deemed negative, so the regional environment was overestimated and overstated. After EC was taken into account, the overestimated environmental depletion assessment results of these counties and cities were corrected.

### 3.2 Comprehensive environmental assessment

#### 3.2.1 EDI

**Fig 8:**
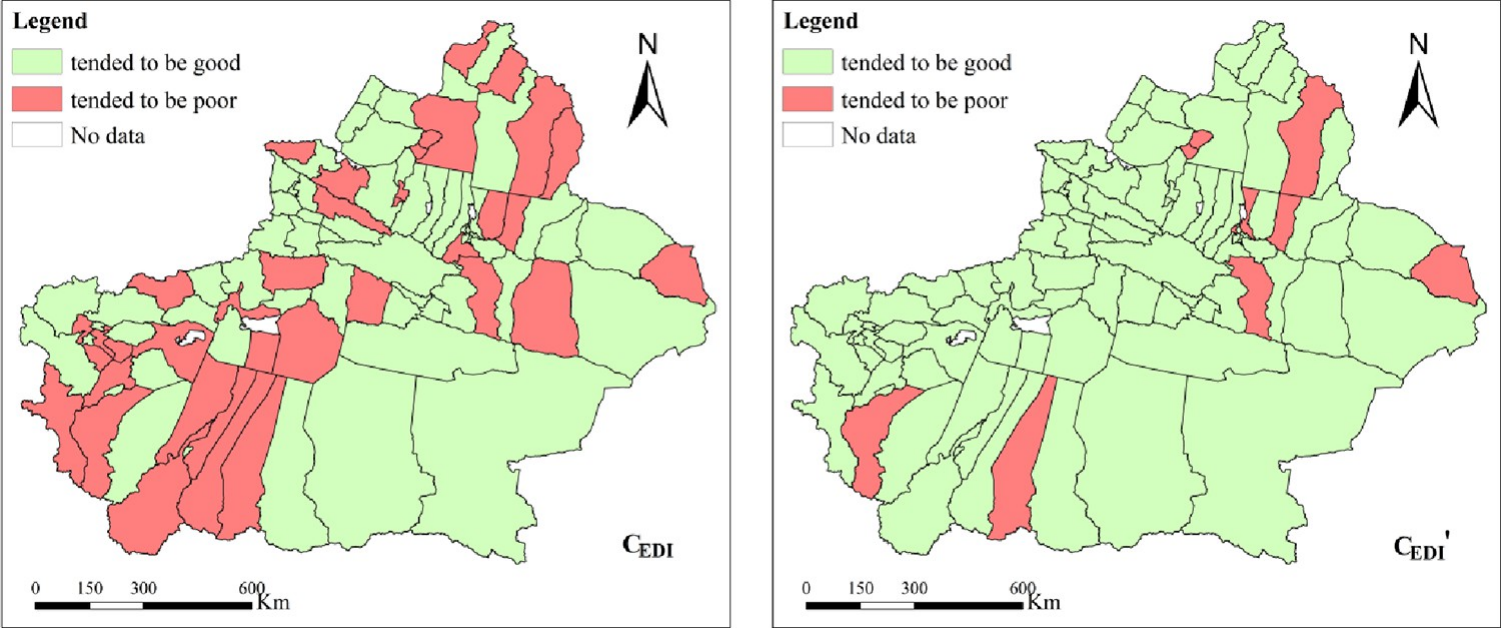
Comprehensive environmental depletion index (C_EDI_) and EDI with EC corrected (C_EDI_’).

According to the single-index evaluation results of COD, NH_3_-N, SO_2_ and NOx based on the EDI, the C_EDI_ categories are obtained according to Table 2, as shown in Fig 8 (Fig 8 –C_EDI_). There were 36 counties and cities whose C_EDI_ tended to be poor, accounting for 38.3%, and 58 counties and cities whose C_EDI_ tended to be good, accounting for 61.70%.

#### 3.2.2 EDI with EC corrected

Based on the single-index evaluation results of COD, NH_3_-N, SO_2_ and NO_x_ modified by EC, the C_EDI_’ categories are obtained according to Table 2, as shown in Fig 8 (Fig 8 –C_EDI_’). C_EDI_’ tended to be poor in 10 counties and cities, namely, Tou Tunhe district, Midong district, Bai Jiantan district, Urhe district, Toksun county, Yiwu county, Jimsar county, Yecheng county, Fuyun county and Yutian county, accounting for 10.64%, and tended to be good in 84 counties and cities, accounting for 89.47%.

#### 3.2.3 Comparative analysis

**Fig 9:**
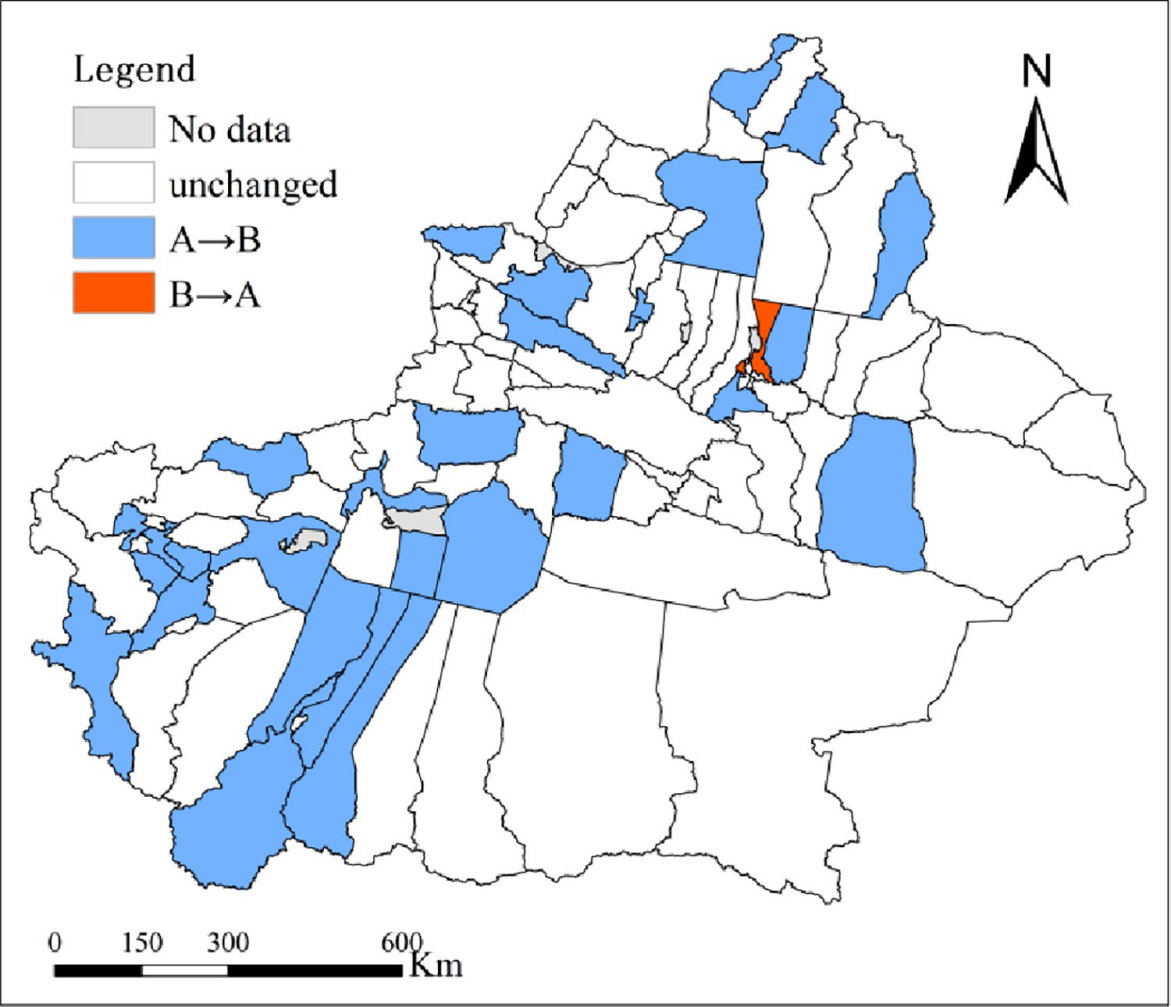
Comparison and visualized analysis for the number of counties and cities by EDI and EDI with EC corrected.

According to section 2.3.2.3, the changes in the number of counties and cities in the comprehensive EDI of the four pollutants with/without considering the EC are shown in Table 4. The category trends of the comprehensive EDI with consideration of EC remained unchanged in 64 counties and cities, accounting for 68.09%, and the trends changed in 30 counties and cities, accounting for 31.91%. The indexes tended to be “good → poor” in 2 counties and cities, namely, Midong district and Tou Tunhe district (Fig 9 –B→A), and tended to be “poor → good” in 28 counties and cities, including Akqi county and Bachu county (Fig 9 –A→B). With consideration of EC, the EDI generally tended to be “good”, which indicated that since 2010, with continuous economic development and expanded environmental governance, the environmental quality in Xinjiang has been gradually improving.

**Table 4:** Comparison of the number of counties and cities by C_EDI_ and C_EDI_’.

Trend	Number of counties and cities	Counties and cities
**Poor → good**	28	Akqi county, Altay city, Bachu county, etc.
**Good → poor**	2	Midong district, Tou Tunhe district
**No change**	64	Akto county, Artux city, Jimsar county, Toksun county, Yiwu county, Yutian county, etc.

### 3.3 Analysis of applicability of methods

In the EDI, the increases in pollutant emissions and economic growth were considered only for environmental assessment, which led to the environment of over 1/4 counties and cities being overestimated or overstated. Hence, it is inevitable that the formulation of environmental policies for regional development will be affected. China explicitly stated in the “Suggestions” that stricter emission permit requirements and policies would be formulated in regions where EC is overloaded and pollutant emissions intensified, which may lead to pollutant emissions quickly increasing in the course of economic development, but counties and cities with minimal pollutant emissions and industrial development lags would implement stricter policies. These may increase economic costs [20], thereby affecting the socioeconomic development of the regions [8, 45]. Furthermore, some counties and cities that have implemented stricter environmental protection policies against high pollution to slow the growth of pollutant emissions but have enormous emissions and severe pollution have been underestimated or unrewarded, leading to the neglect of the pollution conditions of these counties or cities.

The assessment results with consideration of EC indicated that the EDI of 4 kinds of pollutants tended mainly to be good, which indicates that the environmental quality in Xinjiang has been gradually improving since 2010, and environmental pollution control has achieved certain results. Environmental assessment with considering EC is more objective. By assessing the air quality of 338 Chinese cities at the prefecture level and above, Ye et al. [46] found that the air quality index of SO_2_ and NO_2_ in Xinjiang was basically less than 1; that is, Xinjiang experienced low SO_2_ and NO_2_ pollution and had a good environment, consistent with the results reported in this paper. Sun and Zhou [47] discussed the source analysis and spatial distribution characteristics of air pollution in China as well as the relationship between air pollution and exposure risks. The spatial distribution indicated that the SO_2_ and NO_2_ concentrations in Xinjiang were relatively low. By measuring satellite remote sensing data for air quality in Xinjiang using a sampling instrument, Wang et al. [48] found that SO_2_ was distributed mainly on the northern slope of Tianshan Mountain (Xinjiang), Hotan district in South Xinjiang, etc. This finding is relatively consistent with the distribution in the counties and cities where the SO2 intensity tended to be poor. In terms of the water environment, Yan et al. [49] calculated the domestic sewage discharge and pollution load in China and analyzed the spatial distribution of pollutants based on urban water and sewage survey data. They found that the COD emission intensity in Xinjiang was relatively low, indicating that the COD pollution level was low and the water environment quality was good. This finding is relatively consistent with the assessment results considering EC. In addition, these results have been proven in some research. For example, Chen et al. [50] found that COD was not the main water pollutant by quantifying the direct discharge of water pollutants (COD, BOD, etc.) and potential impacts in China in the past 10 years. Zhang et al [23] also found that COD and NH_3_-N pollutants in 89 counties and cities of Xinjiang were not overloaded by assessing the resource and environmental carrying capacity.

A proper balance between economic growth and environmental protection is an important issue for the current transformation under socioeconomic and environmental imbalances [51, 52]. Scientific understanding of environmental assessment can improve the cost-effectiveness of regional air quality management policies and promote a win-win situation for economic growth and environmental protection [9, 53]. Comparative analysis of the environmental assessment results with and without EC shows that the environmental depletion assessment method based on EC correction is more suitable for environmental assessment in Xinjiang. EC, as a bridge that closely links the economic and environmental systems, unifies economic and environmental systems and is a key index for measuring the sustainable development of socioeconomic and ecological environmental systems. We propose to modify the regional EC in the evaluation of the EDI, which makes the evaluation method of environmental depletion universally applicable worldwide and is of great significance to regional environmental evaluation.

## 4 Conclusions

The measurement of changes in environmental depletion is important for the monitoring and early warning of resources and environmental carrying capacity, and objective assessment and knowledge of environmental depletion are important in quickly solving air and water pollution problems and coordinating the sustainable development of regional socioeconomic activities and the ecological environment. The EDI with/without consideration of EC was separately calculated based on air and water pollutant data. The main conclusions are as follows:

1. The EDI aims to reflect changes in regional environmental impact through the ratio relationship between pollutant emission growth and economic growth. However, in terms of pollutant emissions, EC is ignored, rendering the environmental assessment results not objective and reasonable.
2. Comparison of the EDI evaluation with/without considering EC indicates that the environmental assessment results of counties and cities with the EDI evaluation method considering EC show the pollutant emissions increasing rapidly, but the emissions are lower than the EC, which is overestimated. Additionally, pollutant emissions may increase slowly, but emissions may be high and EC underestimated, and these results have been revised. The environmental assessment results are more in line with the objective reality.
3. The evaluation method of the EDI with regional EC correction unifies the economic and environmental systems. It not only provides a scientific basis for the coordinated development of economic, social and environmental systems in Xinjiang but also provides a scientific reference for the sustainable development of the regional economy and environment worldwide. In addition, although the data are operational, data acquisition has certain limitations, and related work needs to be carried out in cooperation with government departments.

## Supporting information

S1 Table Single-index assessment results. (XLS)

S2 Table Comprehensive environmental assessment. (XLS)

S3 Table Comparative analysis. (XLS)

S4 Table Basic data. (XLS)

S1 Appendix (DOCX)
