# Supplementary material for: Discussion of an environmental depletion assessment method–A case study in Xinjiang, China
Source: PLoS One. 2022 Jan 21;17(1):e0262092. doi: 10.1371/journal.pone.0262092 (PMC8782390; doi:10.1371/journal.pone.0262092)
Supplement: S1 Appendix — (DOCX) [file pone.0262092.s005.docx]

# Appendix A

**Table A1 Abbreviations.**

| **Item** | **Descriptions** |
| --- | --- |
| **EDI** | Environmental depletion index |
| **EC** | Environmental capacity |
| **SO2** | Sulfur dioxide |
| **NOx** | Nitrogen oxide |
| **COD** | Chemical oxygen demand |
| **NH3-N** | Ammonia nitrogen |
| **AEDI** | Environmental depletion index of SO2 and NOx |
| **WEDI** | Environmental depletion index of COD and NH3-N |
| **AEDI'** | Corrected environmental depletion index of SO2 and NOx |
| **WEDI'** | Corrected environmental depletion index of COD and NH3-N |
| **PEI** | Pollutant emission growth index |
| **ED** | Economic development index |
| **AEC** | Atmospheric environmental capacity |
| **WEC** | Water environmental capacity |
| **CEDI** | Comprehensive environmental depletion index |
| **CEDI’** | Corrected comprehensive environmental depletion index |
| **NDRC** | National Development and Reform Commission |
| **Technical Method** | Technical Method for the Monitoring and Early Warning of Resources and Environmental Carrying Capacity (Trial) |
| **Suggestions** | Suggestions on Establishing a Long-term Mechanism for the Monitoring and Early Warning of Resources and Environmental Carrying Capacity |
